# Expanding access to postpartum contraception

**DOI:** 10.1097/GCO.0000000000000982

**Published:** 2024-08-02

**Authors:** Michelle Cooper, Kirsten Black, Sharon Cameron

**Affiliations:** a Chalmers Sexual Health Centre, NHS Lothian & Centre for Reproductive Health, University of Edinburgh, Edinburgh, UK; b Department of Obstetrics, Gynaecology & Neonatology, University of Sydney, Sydney, Australia

**Keywords:** contraception, postpartum, service delivery

## Abstract

**Purpose of review:**

Women are particularly vulnerable to unintended pregnancy in the 12 months following a birth. Improving access to postpartum contraception within maternity settings can prevent unintended and closely spaced births, improving the health of mother and child. This review will summarize the recent research in postpartum contraception (PPC), building on existing knowledge and developments in this field.

**Recent findings:**

Current models of postpartum contraceptive provision may not adequately meet women's needs. The COVID-19 pandemic led to changes in postpartum contraceptive provision, with an increasing emphasis placed on maternity services. Antenatal contraceptive discussion is associated with increased postpartum contraceptive planning and uptake of methods after birth. Digital health interventions may be a useful tool to support information about contraception. The most effective long-acting reversible contraceptive (LARC) methods, such as the intrauterine device (IUD) and implant, can be challenging to provide in the maternity setting because of availability of trained providers. Postpartum IUD insertion remains relatively under-utilized, despite evidence supporting its safety, efficacy and cost-effectiveness.

**Summary:**

Antenatal information needs to be partnered with access to the full range of methods immediately after birth to reduce barriers to PPC uptake. Training and education of maternity providers is central to successful implementation of PPC services.

## INTRODUCTION

Fertility and sexual activity can return quickly in the postpartum period [1,2]. Most individuals report that they do not plan to conceive again within the year after childbirth, but pregnancy is not uncommon during this time. A UK study found that 1 in 13 women attend for abortion within the first year postpartum [3]. When conception occurs within a year of childbirth, this short interpregnancy interval places the subsequent pregnancy at an increased risk of complication, including preterm birth, still birth and low-birthweight infants [4]. Therefore, the WHO recommends an interpregnancy interval of at least 24 months to optimize maternal and neonatal outcomes [5]. Most women are unaware of optimal birth spacing [6], and many of the pregnancies that do occur during this time are not planned. In a US study of women who experienced a short interpregnancy interval, around three quarters reported that the pregnancy had been unintended at conception [7]. Unintended and closely spaced pregnancies are known to disproportionately affect those already at the highest risk of unintended pregnancy such as young and socially disadvantaged women [3,4]. This suggests that there is a significant unmet need for effective contraception in the postpartum period, and that current models of care may not adequately meet women's needs. This review will summarize the recent research in postpartum contraception (PPC), building on existing knowledge and developments in this field. 

**Box 1:**
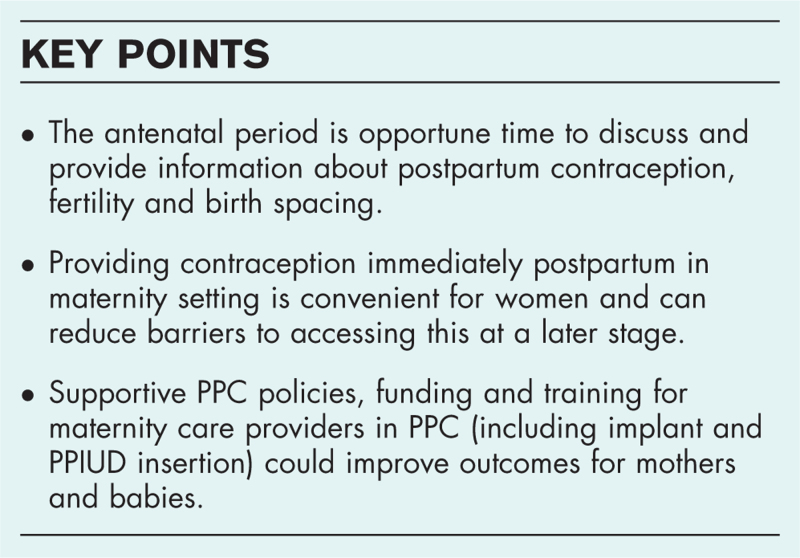
no caption available

## PREVALENCE OF POSTPARTUM CONTRACEPTION

The prevalence of PPC uptake was reported in a systematic review and meta-analysis among women in low-income and middle-income countries that found that across all world regions, the mean contraceptive prevalence rate during the postpartum period was 41.2% [95% confidence interval (CI) 15.7-69.1%]. A low perception of pregnancy risk due to breastfeeding and postpartum amenorrhoea accounted for a lack of contraceptive use [8]. Data from two high-income countries was variable. A US study involving women enrolled in the country's military healthcare system found that 63.3% of women initiated contraception within the first 6 months’ postpartum, most commonly the combined hormonal contraceptive (CHC) methods [9]. In Australian study, using a linked administrative dataset comprising all women who gave birth over a 6-year period in the state of Queensland (*n* = 339, 265 pregnancies) and records of government-subsidized PPC, almost two-thirds (60.2%) did not receive this within the first year after a birth [10^▪▪^].

## SAFETY OF CONTRACEPTIVE METHODS IN THE POSTPARTUM PERIOD

Most contraceptive methods are safe to use during the early postpartum period, that is, first 6 weeks following birth [11] (Table 1). The exception is CHC, which should be avoided during this time because of its higher venous thromboembolism risk and lack of sufficient safety data about the effect on breastfeeding [12,13]. Evidence shows that progestogen-based methods [pill, injectables, implant, levonorgestrel-releasing intrauterine device (IUDs)] do not negatively affect lactation, infant growth or development, and these methods can be safely initiated at any time postpartum [14].

**Table 1:** UK Medical Eligibility Criteria for Contraceptive Use categories applicable to women after childbirth [11]

Condition	Cu-IUD	LNG-IUS	IMP	DMPA	POP	CHC
Postpartum breastfeeding						
0 to <6 weeks	See below		1	2	1	4
>6 weeks to <6 months			1	1	1	2
>6 months			1	1	1	1
Postpartum nonbreastfeeding						
0 to <3 weeks	See below					
With other risk factors for VTE			1	2	1	4
Without other risk factors			1	2	1	3
3 to <6 weeks						
With other risk factors for VTE			1	2	1	3
Without other risk factors			1	1	1	2
>6 weeks			1	1	1	1
Postpartum breastfeeding/nonbreastfeeding						
0 to <48 h	1	1	See above			
48 h to <4 weeks	3	3				
>4 weeks	1	1				
Postpartum sepsis	4	4				

UKMEC	Definition of category
1	Condition where there is no restriction for use of method
2	Condition where advantages of using method generally outweigh the risks
3	Condition where risks generally outweigh advantages of using method. Provision requires expert clinical judgement and/or specialist referral
4	Condition, which represents unacceptable health risk if method is used

CHC, combined hormonal contraception; Cu-IUD, copper intrauterine device; DMPA, depot medroxyprogesterone acetate (progestogen-only injectable); IMP, progestogen-only implant; LNG-IUS, levonorgestrel intrauterine system; POP, progestogen-only pill; UKMEC, UK Medical Eligibility Criteria; VTE, venous thromboembolism.

Although contraception is not strictly required before 3 weeks postpartum, it may be more convenient for women to start their chosen method sooner, especially if it can be provided for them in the maternity setting. Immediate postpartum provision of long-acting reversible methods, such as the implant or IUD, is associated with higher continuation rates and fewer unintended pregnancies in the subsequent year [15–17]. This approach is supported by professional bodies such as the American College of Obstetrics & Gynecology [18] and UK Royal College of Obstetricians & Gynaecologists [12].

## CHANGING MODELS OF POSTPARTUM CONTRACEPTION PROVISION

The ‘traditional’ model of a consultation with a healthcare provider several weeks after birth to discuss PPC, often in primary care, has increasingly been shown to be inadequate. There is often a limited time available for detailed discussion about contraception during the postpartum visit, leading to a focus on shorter acting methods and further additional appointments to initiate a long-acting reversible contraceptive (LARC) [19]. Introducing a discussion about contraception for the first time in the first postnatal days may be suboptimal, as women may be recovering from delivery and preoccupied with caring for the newborn. In addition, given the rapid resumption of fertility and sexual activity, waiting until a 6-week postnatal check may also be too late for some women. In a recent systematic review and meta-analysis, the overall resumption of early sexual intercourse (before 6 weeks) amongst postpartum women was found to be 57% (95% CI 50–64) [20].

These barriers can mean that many women are unable to access their chosen contraception at this time. Around 50% of individuals who plan to initiate LARC at this time, do not attend a follow-up appointment after birth to have this fitted [21]. Many barriers to accessing contraception were exacerbated during the SARS-CoV-2 novel coronavirus (COVID-19) pandemic [22]. In many regions, access to contraception from community settings (such as primary care or sexual health clinics) became more difficult. Some provider-dependent methods, such as sterilization or LARC, also became less widely available.

A retrospective US cohort study of over 600 patients, observed interesting patterns in pre-COVID and during-COVID immediate postpartum contraceptive method provision, that is contraceptive delivery from within the maternity service before discharge compared with delayed or interval several weeks or months later [23]. Higher rates of immediate postpartum LARC were noted in the during-COVID group. There were no differences in the rates of immediate postpartum tubal ligation, but no patients in the during-COVID group received interval tubal ligation in the 6 months after birth. There was a reduction in delayed (or interval) LARC initiation during the pandemic (15–8%; *P* = 0.03). This suggests that women were turning to LARC in the absence of access to sterilization, with increased uptake when this was provided immediately postpartum. This unmet need for postpartum sterilization, driving increased uptake of postpartum LARC methods has also been observed in other studies related to the pandemic [24].

This reflects the unique opportunities the pandemic created for a renewed emphasis on maternity services to provide PPC, as in many cases, this was the only opportunity women had to meet with a healthcare professional to discuss and receive contraception [25]. Some of these initiatives were bolstered by temporary emergency funding and redeployment of staff, raising concerns about sustainability. However, as health services seek to recover from the effects of the pandemic, and with ongoing reductions in global funding for sexual health, many of the challenges to accessing PPC not only persist but also in many cases are more pronounced than before. In the postpandemic world, ensuring adequate antenatal information and access to the full range of methods immediately postpartum, can help to support informed decision-making and reduce the unmet need for PPC.

## ANTENATAL CONTRACEPTIVE INFORMATION

The antenatal period has previously been identified as an opportune time to provide information and discussion about PPC [26], and more recent studies continue to support this. A qualitative study of women having a planned caesarean birth in Sweden found that antenatal contraceptive discussion was welcomed, with most of those interviewed, preferring this discussion during the second half of pregnancy [27]. In a cross-sectional study across two maternity centres in New Zealand, women who recalled both antepartum and postpartum discussion were more likely to have made a contraception plan [odds ratio (OR) 5.6; 2.8–11.5], when compared with those with no recollection of a contraceptive discussion, or postpartum discussion alone (OR 1.77; 1.04–3.02) [28].

Earlier discussion is also associated with increased postpartum contraceptive uptake. In a randomized controlled trial of 280 women with gestational diabetes, the addition of prenatal counselling visit during the third trimester was associated with increased contraceptive use at 6 weeks’ postpartum (87 vs. 55%, absolute risk reduction (aRR) 1.58; CI 1.19–2.10), and greater use of highly effective methods (59 vs. 31%; aRR 1.90; 1.31–2.74) [29]. A recent retrospective review of PPC uptake during the first 6 months of the COVID-19 pandemic, also observed a positive association between prenatal contraceptive planning and contraceptive method uptake after birth [30]. Delivering group-based antenatal information on contraception, for example, during general prenatal classes or for specific pregnant populations such as those with diabetes, has also been linked to increased uptake of postpartum LARC [31,32].

Conversely, inadequate knowledge amongst women about postpartum contraceptive methods and their safety continues to be a barrier to uptake [33,34]. Recent studies from both low-income and high-income settings have reported a particular lack of knowledge around lactational amenorrhoea method, for example, its necessary criteria for effectiveness (amenorrhoea, exclusive breastfeeding, less than 6 months’ postpartum). Information about this method is also often omitted from clinician PPC counselling [35,36].

Autonomous decision-making is central to quality care, especially for contraceptive methods that require a provider and at more vulnerable times in life, such as during the postpartum period [37]. In a retrospective survey study, evaluating the quality and provision of contraception counselling to pregnant women with cardiac disease, 65% reported generally high satisfaction with their provider discussion [38]. Respondents particularly valued thorough counselling and respect for their autonomy during these discussions, with dislike of feeling ‘pressured’. The way in which information is provided, therefore, continues to be explored, including the role of digital health interventions (DHIs), which have already shown promise in the nonpostpartum context [39^▪▪^].

## DIGITAL HEALTH INTERVENTIONS

A 2023 systematic review of the effectiveness of DHI specific to the postpartum context, concluded that different modalities had the potential to increase PPC use to different degrees, but that the impact of these interventions on repeat pregnancy rates remained unclear [40^▪^]. A qualitative study from India recently evaluated a DHI (consisting of a mobile application, virtual meetings, and group text) amongst postpartum women in India, with respect to acceptability and perceived impact [41]. Users were highly satisfied and reported an increased motivation towards positive health behaviours including PPC initiation. However, several barriers to ‘scaling up’ were also identified, including technical difficulties and a lack of internet connectivity amongst the wider population.

Equitable access is an important factor to consider as the potential of DHI continues to be explored, and meaningful engagement and co-design with users is central to this. This approach was employed successfully to develop a recent PPC animation in the UK, which provided key facts on return of fertility after delivery, when contraception can be safely initiated and available methods [42]. The article describes the approach to co-design, and the opportunities this communication modality can offer to enhance accessibility and inclusivity.

However, digital media also has the potential to convey misinformation. A content analysis of information about lactational amenorrhoea on one online video platform reported high rates of false or incomplete information, despite the high levels of viewer engagement, indicating an interest in this method [43]. Therefore, the role of healthcare professionals remains critical. They should be able to confidently and accurately discuss the advantages and disadvantages of all methods to help dispel misinformation and support informed choice.

## TRAINING AND EDUCATION OF PROVIDERS

Several recent studies have shown that staff working in postpartum settings may lack accurate knowledge about PPC [44]. In a survey of 289 midwives providing postpartum care in Australia, two-thirds indicated a lack of formal contraceptive training or education in their role [45]. A study from the UK found that 74% of health visitors (community public health nurses who provide support for families and infants in the first few years after birth) reported receiving no training in PPC [46]. This lack of knowledge and training can lead to misconceptions about the suitability and safety of certain methods, which can further restrict patient access to effective contraception.

A recent interview-based study of maternity care providers in Australia, recognized the importance of shared responsibilities and collaboration between different healthcare professionals involved in pregnancy and postnatal care, to provide a consistent and co-ordinated approach to PPC delivery [47]. Diversifying the cadre of staff trained to provide contraceptive information or supplies, can help to expand existing opportunities to women to access PPC. For instance, in one US study of 263 women, attending paediatric well child clinics in the first 6 months postpartum, around a quarter had an ongoing unmet need for contraception [48]. Most were supportive of receiving contraceptive information and supplies in this setting. Linking contraceptive provision with infant vaccination visits was also found to be a feasible and acceptable strategy amongst stakeholders in rural India [49].

However, in order to be fully effective in reducing barriers, contraception discussion needs to be partnered with rapid and simplified provision of methods in the immediate postpartum setting. It can be especially challenging to provide LARC in maternity settings, as these require the ‘on demand’ availability of a trained provider, and many clinicians are unfamiliar with techniques such as immediate postpartum intrauterine device insertion (PPIUD) [50]. This is one reason why this PPIUD is relatively under-utilized in most parts of the world. In a recent survey of contraception experts from 29 European countries, just over half reported that any contraceptive methods were provided in maternity settings in their country, with even lower rates of immediate postpartum LARC provision [51]. Some of the reported barriers included: cost, lack of policy or government support, lack of awareness and training for maternity staff.

## ACCESS TO IMMEDIATE POSTPARTUM LONG-ACTING REVERSIBLE CONTRACEPTIVE

Immediate PPIUD insertion is a well tolerated and effective technique [52]. A 2023 systematic review reported high 6-month and 12-month continuation rates of PPIUD (over 80%) amongst the 133 included studies [53^▪▪^]. However, one of the perceived drawbacks of PPIUD insertion is the higher rate of device expulsion compared with insertion in the nonpostpartum period. There is also a higher expulsion rate with observed vaginal versus caesarean PPIUD insertion [52,53^▪▪^]. PPIUD has been shown to remain cost effective up to expulsion rates of 56% [54], but research continues into factors, which may reduce the rate of this outcome, including innovations related to the insertion method itself.

A dedicated PPIUD inserter (one that is longer and wider than a standard IUD inserter to accommodate the larger postpartum uterus) has shown promise compared with conventional forceps-assisted insertion of vaginal PPIUD, with similar rates of expulsion and complications but higher satisfaction amongst providers [55]. More recently, this inserter has been also evaluated for use during caesarean [56]. In a randomized controlled trial of over 800 PPIUD recipients in Egypt, a significant reduction in IUD displacement was observed between conventional manual placement and the new inserter at 6 weeks (15.5 vs. 9.9%; *P* = 0.019) and 6 months (20.1 vs. 11.9%; *P* = 0.002). The incidence of nonvisible threads was also reduced in the inserter group (9.6 vs. 3.6%; *P* = 0.001).

Fundal placement of PPIUD has long been linked to a reduction in the expulsion risk. The ability to place the IUD at or near the fundus is also an indirect measure of the competence and experience of the provider, particularly for vaginal PPIUD insertion, which is a ‘blind’ technique. A recent study from the United States evaluated the first 3 years of a PPIUD training programme and observed a reduction in the service-wide expulsion rate over time, from 28% in year 1 to 15% in year 3 (*P* = 0.03) [57^▪^].

Other newly established services have observed a high initial expulsion rate [58], and more longitudinal studies are needed to evaluate this. But existing data supports the need for robust and evidence-based training in PPIUD insertion. In its absence, a higher than expected expulsion rate may be observed, jeopardising the ‘buy in’ necessary from patients and other key stakeholders to initiate and sustain PPIUD services.

Immediate insertion of implant is also well tolerated, effective and does not impact breastfeeding outcomes [14–16]. There is evidence to support the feasibility and acceptability of midwives inserting contraceptive implants in women's homes [59,60]. This can overcome barriers to access this method for those who are unable to receive this from maternity hospital.

Many of the difficulties in introducing LARC to existing models of maternity care are related to the external environments in which it is provided, rather than the methods themselves. This has led to an increasing interest in the role of implementation science in PPC services more broadly [61]. To fully realise the public health benefits of improving access to PPC, and reduce global disparities in access, further investigation into these systemic facilitators and barriers is needed [53^▪▪^]

## CONCLUSION

There is a high unmet need for contraception in the postpartum period. To provide truly holistic care, women need to be provided with accurate information in the antenatal period, in a range of formats to suit their individual needs. This needs to be partnered with rapid and simplified access to the full range of methods in the immediate and early postpartum setting, including availability of under-utilized techniques such as PPIUD insertion, ideally at no or low cost to patients. This can be achieved through investment in tailored training and education programmes for all staff working with individuals during and after pregnancy, through a whole systems approach. As maternity services seek to maintain safe care in the face of increasing clinical complexity and workforce pressures, there is a need to reframe PPC provision, beyond its clear role in reproductive autonomy, as a positive intervention for wider maternal and neonatal health outcomes.

## Acknowledgements

*None.*

### Financial support and sponsorship

*None.*

### Conflicts of interest

*There are no conflicts of interest.*

## REFERENCES AND RECOMMENDED READING

Papers of particular interest, published within the annual period of review, have been highlighted as:

▪ of special interest
▪▪ of outstanding interest
